# Early Rising

**Published:** 1884-06

**Authors:** 


### EARLY RISING.

THE proper time to arise is when sleep, properly so-called, ends. Dozing is not admissible from any reasonable or health point of view. The brain falls into a state we call sleep, and the other organs of the body follow it. True sleep is the aggregate of sleeps. In other words, sleep, which must be a natural function—*i. e.*, physiological instead of pathological, or induced by disease or drugs—is a state which consists in the sleeping or rest of all the several parts of the organism. Sometimes one and at other times another part of the body as a whole may be the least fatigued and so the first to awake, or the most exhausted, and therefore the most difficult to arouse. The secret of a good sleep is—the physiological conditions of rest being established—to so work and weary the several parts of the organism as to give them a proportionally equal need of rest at the same moment. The cerebrum or mind organ, the sense organs, the muscular system, and the viscera should be all ready to sleep together, and, so far as may be possible, they should be equally tired. To wake early and feel ready to rise, this fair and equal start of the sleepers should be secured; and the wise self-manager should not allow a drowsy feeling of the consciousness or weary senses, or an exhausted muscular system, to beguile him into the folly of going to sleep again when once his consciousness has been aroused. After a very few days of self-discipline the man who resolves not to "doze" that is, to allow some still sleepy part of his body to keep him in bed after his brain has once awakened—will find himself, without knowing why, an "early riser."—*Lancet.*

Many have become victims to the use of opium and morphine from the administration of these drugs for the relief of neuralgia. It is very gratifying to observe that such dangerous consequences may be averted by the use of Tongaline, or "Liquor Tongae Salicylatus," which is almost a specific in the acute forms of that complaint.—*Extract from June number of Medical Brief.*

We take pleasure in calling attention to the Ladies' Protector and Supporter. It is a new and novel appliance of the utmost value to every lady, supplying a long-felt want. It is an article of superior merit invented and patented by Madame L. Lange of New York City Its claims are fully and fairly set forth in the descriptive circular referred to in the advertisement in this issue.

